# Spatiotemporal transcriptome provides insights into early fruit development of tomato (*Solanum lycopersicum*)

**DOI:** 10.1038/srep23173

**Published:** 2016-03-18

**Authors:** Shuaibin Zhang, Meng Xu, Zhengkun Qiu, Ketao Wang, Yongchen Du, Lianfeng Gu, Xia Cui

**Affiliations:** 1 Key Laboratory of Biology and Genetic Improvement of Horticultural Crops of the Ministry of Agriculture, Sino-Dutch Joint Laboratory of Horticultural Genomics, The Institute of Vegetables and Flowers, Chinese Academy of Agricultural Sciences, Beijing 100081, China; 2 Haixia Institute of Science and Technology (HIST), Fujian Agriculture and Forestry University, Fuzhou 350002, China

## Abstract

Early fruit development is crucial for crop production in tomato. After fertilization, the ovary undergoes cell division and cell expansion before maturation. Although the roles of regulatory signals such as hormone and carbohydrate during early fruit development have been studied, the spatial distribution and the sequential initiation of these regulatory signals still need to be explored. Using the tomato cultivar ‘Moneymaker’, we analyzed the transcriptome of the ovule and the ovary wall/pericarp dissected from four different stages of the early developing fruits by stereoscope. These datasets give us the whole picture about the spatial and temporal signal distribution in early development of ovule and pericarp. Our results indicate that the hormone signal was initiated in both ovule and pericarp after fertilization. After that, different signals were activated in ovule and pericarp due to their distinct developmental processes. Our study provides spatiotemporal regulatory landscape of gene expression with sequential information which was not studied by previous work and further strengthens the comprehension of the regulatory and metabolic events controlling early fruit development.

Tomato is an ideal model system for fleshy fruit development because of its rapid life cycle, the economic importance and the availability of genome information. A tomato fruit is derived from the ovary after anthesis and successful fertilization. The ovary wall forms the pericarp of the fruit and the ovules develop to seeds.

The observation on a wild tomato species *Solanum pimpinellifolium* LA1589 showed the ovule and the ovary wall undergo different developmental processes^1^. After fertilization, the embryo in the ovule goes through a cell division period and reaches to a 4–16 celled embryonic stage at approximately 3–6 days post anthesis (DPA). Meanwhile, the cells in ovary wall start to divide at 2 DPA. At the time around 5 DPA, the cell division in ovary wall is nearly ending and the cell expansion period begins^1^. No clear boundary exists between the cell division and cell expansion stages during pericarp development, the end of cell division is overlapped with the beginning of cell expansion^2^. The early developmental stage is crucial for the fruit formation. First, whether the fruit development will proceed or abort is decided shortly after anthesis depending on fertilization. Second, the size or weight of fruit is largely dependent on the cell number of the pericarp which is determined during cell division stage. By now, two genes affecting fruit size, *FW2.2* and *FW3.2*, have been cloned and are considered to affect fruit size by regulating cell division at early fruit developmental stages^3^,^4^.

In recent years, many efforts have been made to unravel the regulatory mechanism during early fruit development of tomato. The phytohormones including auxin, gibberellins (GAs) and cytokinin play important roles during early stages of fruit formation^5^. Auxin and GAs are not only the major positive signals regulating fruit initiation but also important for stimulating cell division or inducing cell expansion, respectively^6^. Cytokinin regulates fruit development partially dependent on the metabolism of auxin and GAs^7^. Moreover, rapid cell division and expansion depend on sufficient energy supply. During early fruit development, the soluble sugars as well as starch accumulated in the pericarp^8^,^9^. When plants are under stress conditions which block sugar import, the insufficient energy supply will trigger programmed cell death and lead to the abortion of fruit development^10^. Although the importance of hormone and carbohydrate signals on regulating early fruit development is well known, the spatial distribution and the temporal expression of the regulatory genes are still poorly understood. Recent works showed elevated expression of auxin biosynthesis genes in ovule tissues of 4 DPA tomato fruit^11^ and auxin was predominantly synthesized in developing embryo and so-called ghost tissue including endosperm and seed coat in strawberry^12^. Compared with strawberry fruit, the ovary wall formed the pericarp in tomato as the main part of the fleshy fruit, whereas such tissue is barely developed in strawberry. Thus it is necessary to clarify the dynamic regulation in tomato early fruit development.

Several studies have tried to unravel the regulation network of early fruit development through large scale transcriptomic analysis in tomato^13^,^14^,^15^. However, these works lack either tissue specificity or dynamic signaling changes during this process. A recent study dissected diverse tissues in early developing fruits of wild tomato species LA1589 at 0 DPA and 4 DPA by laser-capture microdissection (LCM) and presented tissue-specific expression profiles of genes regulating the transition from ovary to fruit^11^. Nevertheless, their analysis lacks the sequential information of different regulatory signals during fruit development after fertilization. Here, we collected the ovule and ovary wall/pericarp from tomato cultivar ‘Moneymaker’ dissected under stereoscope at 1 DPA, 2 DPA and 5 DPA which reflected to the time just after fertilization, the beginning of cell division and the overlapping time point of cell division and cell expansion respectively. The comparative analysis of their transcriptomes reveals that the early development of tomato fruit establishes a temporal expression gradient of genes which correlates to cell division and embryo formation. Our results provide a model elucidating the spatial and temporal regulation of early fruit development in tomato.

## Results

### RNA-seq analysis of ovule and ovary wall in different developmental stages

**Figure 1:**
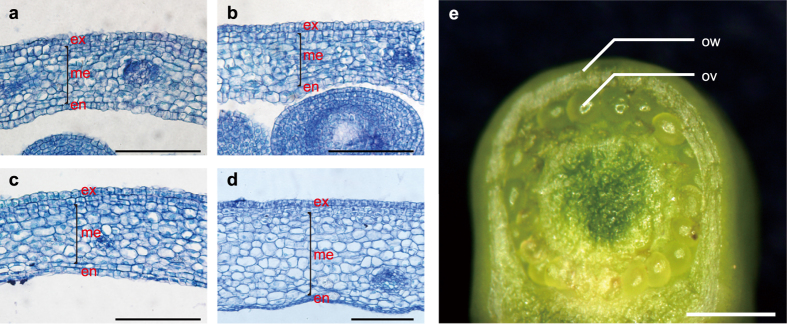
The materials used for RNA-seq. **(a–d)** The microscopy images of paraffin embedded tissues of 0 DPAe (**a**), 1 DPA (**b**), 2 DPA (**c**) and 5 DPA (**d**) fruit showing the cell layer in ovary wall/pericarp. Scale bars = 100 μm. ex, exocarp; me, mesocarp; en, endocarp. **(e)** The ovule and the ovary wall/pericarp samples collected for RNA-seq using a fruit of 0 DPAe as an example. Scale bar = 0.5 mm. ov, ovule; ow, ovary wall/pericarp.

To analyze the transcriptomes of ovule and ovary wall in early developing fruits, we collected the ovaries of tomato cultivar ‘Moneymaker’ at 1 DPA, 2 DPA and 5 DPA. As a control which represented the time just before fertilization, we emasculated some flowers at –2 DPA and collected the same tissue at 0 DPA, which were noted as 0 DPAe. The microscopy images of paraffin embedded tissues showed that there are more cell layers in ovary wall, especially in exocarp and endocarp of 2 DPA than that of 0 DPAe and 1 DPA (Fig. 1a–c) suggesting that the cells in ovary wall started to divide at 2 DPA. The ovary wall significantly thickened at 5 DPA (Fig. 1d). Besides that, the cell volume in mesocarp of 5 DPA increased (Fig. 1d), indicating that 5 DPA was a time point when cell expansion started to occur.

For the ovaries of four developmental stages, we dissected the ovule and ovary wall under stereoscope (Fig. 1e). A total of eight samples with two biological replicates for each sample were collected and the RNA was extracted to construct RNA sequencing libraries and sequence with a paired-end 125 bp sequencing strategy. In total, about 356 million (356,040,398) clean fragments were generated and more than 88% of them could be mapped to the *S. lycopersicum* genome^16^. The unique fragments which occupied more than 97% of the mapped fragments in all samples were used for the following analysis (Supplementary Table S1). The expression of genes was normalized as fragments per kilobase of transcript per million mapped reads (FPKM, Supplementary Table S2).

### Identification of differential expressed genes during early fruit development

**Figure 2:**
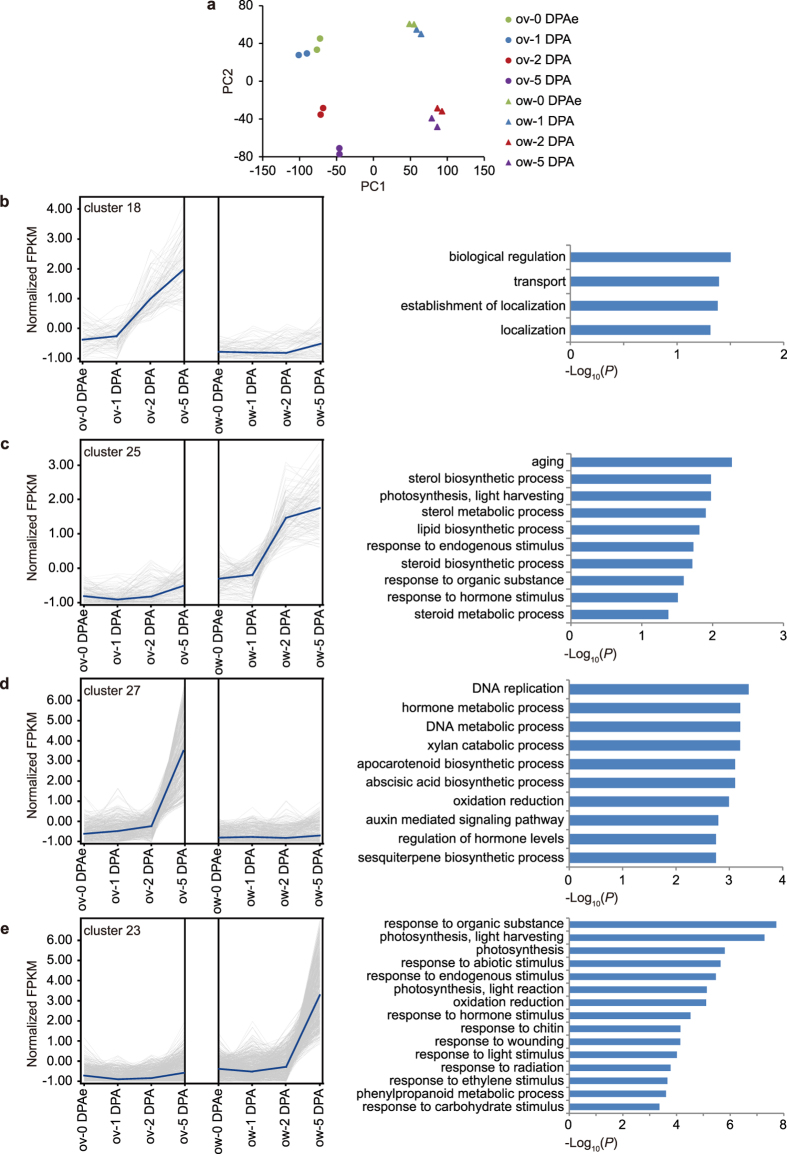
Co-expression analysis of differential expressed genes. (**a)** Principle component analysis (PCA) of the RNA-seq datasets. The circles indicate the ovule samples and the triangles indicate the ovary wall/pericarp samples. Green, blue, red and purple indicate samples from 0 DPAe, 1 DPA, 2 DPA and 5 DPA respectively. (**b–e**) The co-expression pattern (left) and the enriched GO terms in biological processes (right) of cluster 18 (**b**), 25 (**c**), 27 (**d**) and 23 (**e**). The grey lines show the normalized FPKM of individual genes, the blue lines indicate the average of all clustered genes. ov, ovule; ow, ovary wall/pericarp.

To assess the repeatability of our experiments and the similarity among our datasets, we performed a principal component analysis (PCA). As the results showed, the two replicates of each sample located nearest with each other. Our further statistical analysis showed higher correlations between these two replicates (Supplementary Table S1), demonstrating the good repeatability between them, which reflected reliability of our datasets. The ovule samples were apart from the ovary wall samples indicating the distinct expression profiles in ovule and ovary wall/pericarp. The datasets from 1 DPA were similar with those from 0 DPAe suggesting that the fertilization signals were just generated and did not alter the expression profiling in both ovule and ovary wall/pericarp significantly at 1 DPA. The samples of 2 DPA and 5 DPA were far from 1 DPA indicating that fertilization changed the expression profiling of both ovule and ovary wall/pericarp dramatically (Fig. 2a).

We considered the 0 DPAe ovaries which were at the time just before fertilization to be the start point of fruit development and compared other samples with the same tissues of 0 DPAe respectively. Only genes with both fold change >3 and the adjusted *P* value < 0.001 in the comparisons were identified as differential expressed genes (DEGs). Using this rigorous cutoff, we identified 780, 1,728 and 2,746 DEGs in the ovules of 1 DPA, 2 DPA and 5 DPA compared with 0 DPAe respectively. In the ovary wall/pericarp, 738, 2,819 and 2,691 DEGs were identified within the comparisons of same periods. To explore the DEGs in a time-series way, we also performed sequential pairwise comparisons among 1 DPA, 2 DPA and 5 DPA in both ovule and ovary wall/pericarp. In ovule, there were 1,738 DEGs between 1 DPA and 2 DPA or 2,353 DEGs between 2 DPA and 5 DPA. In ovary wall/pericarp, 2,142 and 2,301 DEGs were identified within the same comparisons of periods. In a total, 7,455 DEGs were found during ovule and ovary wall/pericarp development (Supplementary Table S3). A heatmap using these DEGs indicated that these DEGs displayed tissue-specific and temporal expression pattern in ovule and ovary wall/pericarp (Supplementary Fig. S1). Among all these DEGs, there were 646 transcription factors (TFs) belonging to 48 families according to Plant Transcription Factor Database (http://planttfdb.cbi.pku.edu.cn/index.php?sp=Sly)^17^ (Supplementary Fig. S2a, Supplementary Table S4). GO analysis showed that these differential expressed TFs were enriched in response to hormone stimulus, response to carbohydrate stimulus, post-embryonic development and so on (Supplementary Fig. S2b), indicating the important roles of phytohormone and carbohydrate signals during early fruit development.

Different with our work using four distinct developmental stages of pericarp and ovule, a recent work explored the transcriptomes of diverse tissues in tomato fruits at 0 DPA and 4 DPA by LCM coupled with RNA-seq in a wild species (LA1589), and provided high resolution of fruit and seed tissue-specific regulatory framework^11^. Comparing these two datasets will help us to understand early fruit development comprehensively. Thus, we analyzed the DEGs in pericarp which was the only relative identical tissue in two studies. We compared DEGs of 4 DPA in the published dataset and 5 DPA in our work using 0 DPA and 0 DPAe as control, respectively, since these stages were similar and comparable. There are 883 DEGs in pericarp overlapped in these two dataset (Supplementary Fig. S3a). A considerable proportion of DEGs in these two experiments were different, such as 151 TFs only existed in our results and 229 TFs specifically detected in the published dataset (Supplementary Table S5), suggesting that these two studies provide different regulatory map of gene expression during early fruitdevelopment. Different genetic background of cultivated tomato and wild species may cause inconsistence of fruit developmental processes in these two species. In addition, the developmental stages used for comparison are not identical although they are very similar. Besides that, the RNA obtained by LCM had to be amplified before library construction which would cause strong bias of read abundances towards the 3′ end (Supplementary Fig. S3b). Therefore, these two works complement each other and will help for understanding early fruit development of cultivated tomato and wild species.

We further analyzed the dynamic transcriptomes at four developmental stages in two distinct tissues. The DEGs were clustered into 37 groups according to their expression profiles in different tissues and stages (Supplementary Fig. S4 and Supplementary Table S6) and we focused on genes showing distinct spatiotemporal expression pattern. The Cluster 18 contained genes showed elevated expression after fertilization at 2 DPA specifically in ovule and maintained high expression level at 5 DPA (Fig. 2b). Genes in this cluster were predominantly enriched in biological regulation and transport based on GO analysis (Fig. 2b). Genes in cluster 25 expressed mainly in ovary wall/pericarp with a similar temporal trend of cluster 18 (Fig. 2c), GO terms enriched in this cluster included response to hormone stimulus (Fig. 2c), suggesting that the importance of phytohormone at the beginning of pericarp development after fertilization. Different with these two clusters, genes in cluster 27 and cluster 23 had expression peak at 5 DPA and preferentially expressed in ovule or ovary wall/pericarp, respectively (Fig. 2d,e). GO analysis of these two clusters revealed that xylan catabolic process and response to carbohydrate stimulus was enriched respectively (Fig. 2d,e), suggesting that sugar metabolism was becoming important at 5 DPA fruit. Besides that, the genes of cluster 27 were also assigned in several GO terms including DNA replication (Fig. 2d). In cluster 23, genes were enriched in photosynthesis (Fig. 2e) in line with previous proteomic data that the photosynthesis related proteins increased during cell expansion period^18^. Taken together, these results indicate that dynamic regulation context is established during pericarp and embryonic developments.

### Specific expression pattern in ovule and ovary wall

**Figure 3:**
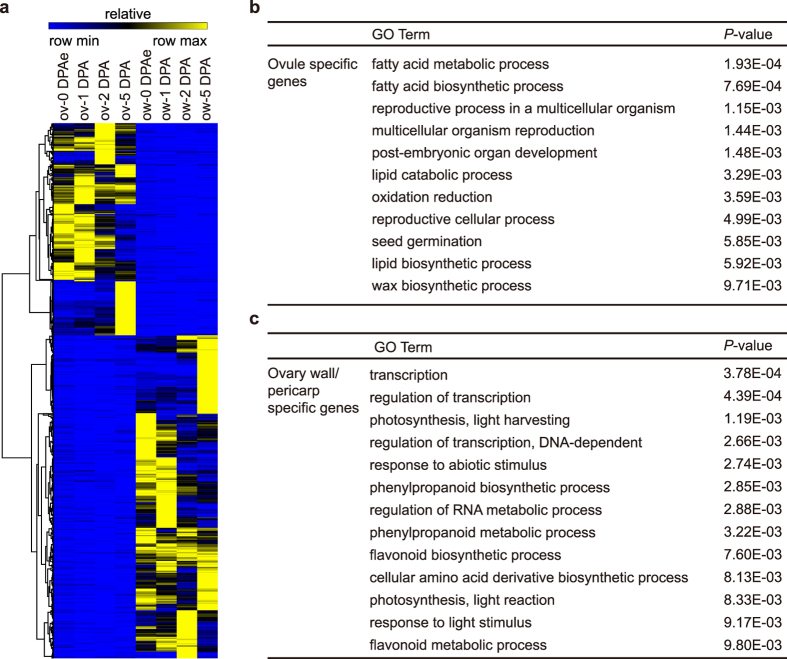
Identification of ovule and ovary wall/pericarp specific expressing genes. (**a)** The relative expression pattern of ovule and ovary wall/pericarp specific expressing genes. **(b)** The enriched GO terms in biological processes of ovule specific expressing genes. **(c)** The enriched GO terms in biological processes of ovary wall/pericarp specific expressing genes. ov, ovule; ow, ovary wall/pericarp.

Since ovule and ovary wall/pericarp showed distinct expression profiles, we identified the genes specifically expressed in ovule or ovary wall/pericarp. We defined the genes with FPKM < 1 in all ovary wall/pericarp samples and FPKM ≥ 3 in any of the ovule samples as ovule specific genes and *vice versa*. Using this filter condition, we identified 283 genes specifically expressed in ovule and 431 genes in ovary wall/pericarp (Fig. 3a and Supplementary Table S7). We selected several genes for RT-qPCR validation and the results showed significant specificity as the RNA-seq data presented (Supplementary Fig. S5). To explore the possible functions of tissue-specific genes, GO analysis was performed. The genes specifically expressed in ovule were assigned to GO terms including fatty acid metabolic process, lipid catabolic process and oxidation reduction (Fig. 3b). Among GO terms enriched significantly in ovary wall/pericarp specific genes, transcription and photosynthesis had a high ranking position (Fig. 3c). Besides that, genes involved in phenylpropanoid and flavonoid metabolic process were also enriched in ovary wall/pericarp specific genes indicating the specific metabolic events during pericarp development (Fig. 3c).

Previous results indicated that phytohormones, especially auxin, GAs and cytokinin play essential roles in tomato fruit formation and early development^5^. A recent work exhibited a high spatial resolution of tissue-specific expression pattern of genes related with different hormones^11^. To obtain the detailed expression profiles of hormone related genes with developmental dynamics of ovary wall/pericarp, we determined the expression pattern of these hormone related genes involved in the metabolism and signalings of auxin, GAs and cytokinin^19^,^20^,^21^,^22^,^23^ (Supplementary Table S8). TAA/TARs and YUCCAs are two important enzyme families constituting the predominant biosynthesis route of auxin. Previous studies indicated that auxin is mainly synthesized in embryo and is transported to pericarp to promote its development^11^,^24^. In our result, *ToFZY2* (*Solyc08g068160*), *ToFZY3* (*Solyc09g091090*) and a TAR family member *Solyc03g112460* were mainly expressed in ovule (Supplementary Fig. S6a). However, we found that *ToFZY1* (*Solyc06g065630*) and *ToFZY5* (*Solyc06g083700*) were specifically expressed in ovary wall during early fruit developmental stages (Supplementary Fig. S6a). In addition, the expression level of *ToFZY5* was enhanced dramatically in ovary wall/pericarp of 2 DPA fruit (Supplementary Fig. S6a). This result suggested that the auxin may be synthesized in the ovule and the ovary wall/pericarp independently during early fruit development. We further validated the specific expression of *ToFZY2* and *ToFZY1* by RT-qPCR. Consistent with our RNA-seq results, *ToFZY1* was expressed specifically in pericarp at a relative stable expression level and the transcription of *ToFZY2* was increased gradually after fertilization in ovule at a temporal regulation manner (Supplementary Fig. S7).

Not only auxin biosynthesis, the auxin transport also takes part in regulating spatiotemporal auxin signals during fruit development. Our observation demonstrated that several auxin transport genes including *PIN* and *AUX/LAX* family members were expressed in a spatial-temporal manner. During fruit development, *PIN5* (*Solyc01g068410*) was expressed specifically in ovule and the expression level was elevated gradually after fertilization (Supplementary Fig. S6b and S7). On the contrary, *PIN6* (*Solyc06g059730*), *PIN1* (*Solyc03g118740*), *PIN7* (*Solyc10g080880*) and *LAX3* (*Solyc11g013310*) were mainly expressed in ovary wall/pericarp with a decrease after fertilization (Supplementary Fig. S6b and S7). We also analyzed the global expression profiles of all *ARF*s and *Aux*/*IAA* genes which are the two main families of auxin responsive genes and clustered genes with similar expression pattern together (Supplementary Fig. S6c). *ARF7* (*Solyc07g042260*) which was reported to negatively regulate fruit set^25^ had a peak expression in ovule at 0 DPAe and its expression decreased after fertilization (Supplementary Fig. S6c). *ARF9* (*Solyc08g082630*) was expressed in both ovule and ovary wall/pericarp but showed a time-course expression pattern which was elevated with fruit development (Supplementary Fig. S6c) coincide with previous work^26^. Besides that, we observed that *IAA14* (*Solyc09g083290*) and *IAA35* (*Solyc07g008020*) were highly expressed in ovule at 5 DPA and *IAA17* (*Solyc06g008590*) specifically expressed in ovary wall/pericarp indicating their potential role in embryo or pericarp development respectively (Supplementary Fig. S6c and S7).

Besides auxin, several GA and cytokinin related genes also displayed spatiotemporal expression pattern during early fruit developmental stages (Supplementary Fig. S8 and S9). Compared with hormone signaling genes, the hormone metabolism genes showed more specificity among different tissues and stages suggesting that *in situ* hormone biosynthesis may be important for early fruit development.

### Spatial and temporal expression pattern of transcription factors

**Figure 4:**
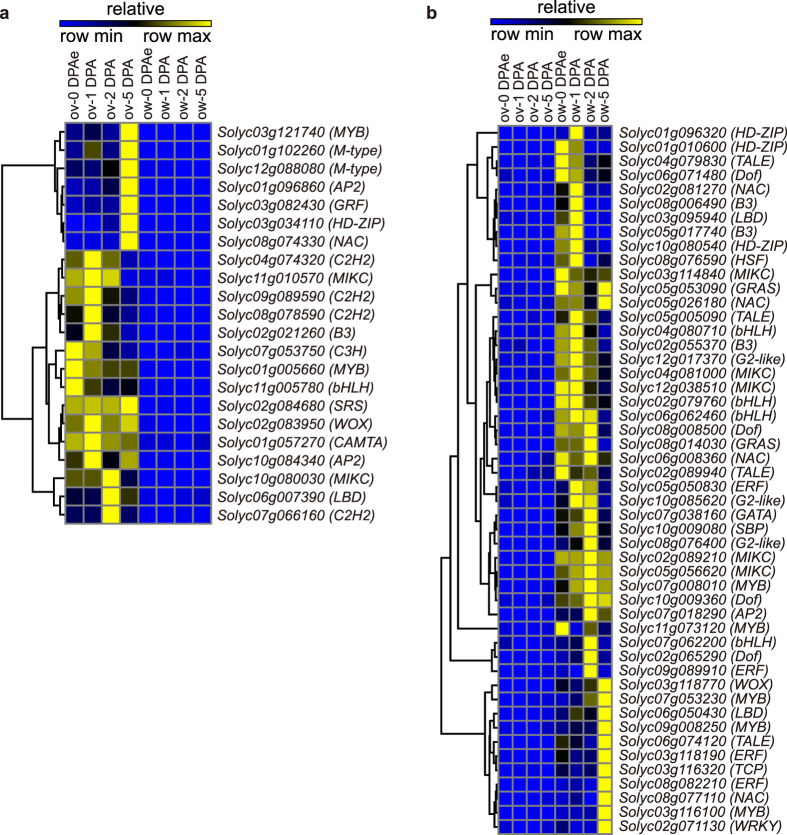
The relative expression pattern of tissue specific transcription factors. (**a)** The relative expression pattern of ovule specific transcription factors. **(b)** The relative expression pattern of ovary wall specific transcription factors. ov, ovule; ow, ovary wall/pericarp.

Intensive studies have revealed that TFs play vital roles in regulating gene expression as repressors or activators in all living organisms and are involved in various important cellular processes, such as development, hormone response, environmental adaptability and immunity^27^,^28^. As a large number of genes involved in transcriptional regulation specifically expressed in ovule and ovary wall/pericarp, we further analyzed the TFs specifically expressed in ovary wall/pericarp or ovule during fruit development. Fifty TFs belonging to 19 families were only expressed in ovary wall/pericarp and 22 TFs of 15 families showed specificity in ovule at early fruit developmental stages (Supplementary Table S9). One of the enriched TF families in ovule was M-type family which belongs to a subgroup of MADS-box gene family^29^. M-type TFs are believed to take part in plant reproductive regulation particularly during embryo and endosperm development^30^, which could be reflected from our results that most specifically expressed M-type TFs showed elevated expression at 5 DPA in ovule (Fig. 4a). Besides that, two TFs of AP2 family were also expressed specifically in ovule (Fig. 4a). The AP2 members in *Arabidopsis* were reported to function in embryo development and organ primordial formation^31^,^32^ suggesting the conserved functions of this family among different plant species. Remarkably, TALE family contained the most ovary wall specific members within tomato genome (4/27) (Fig. 4b, Supplementary Table S9). The TALE family members were found to play important roles in carpel development in *Arabidopsis*^33^. This indicated that the role of regulating reproductive organ of TALE family members may be conserved across species. Thus our results undoubtedly provide new candidates of TFs for exploring their functions in early fruit development.

### Expression profiles of sugar related genes in different tissues and stages

**Figure 5:**
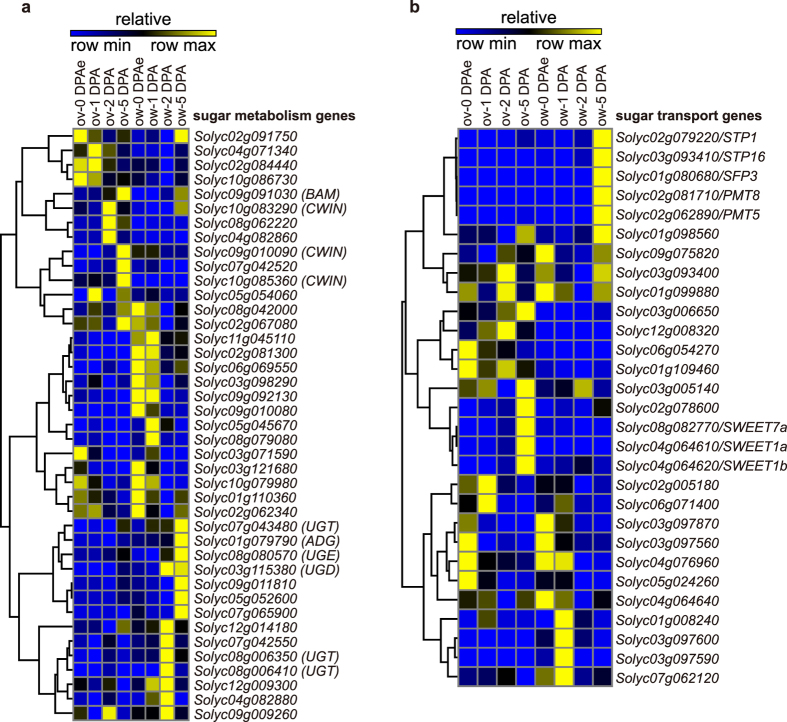
The relative expression pattern of sugar related genes. (**a)** The relative expression pattern of differential expressed sugar metabolism genes. BAM, CWIN, UGT, ADG, UGE and UGD indicate beta-amylase, cell wall invertase, UDP glycosyltransferase, ADP glucose pyrophosphorylase, UDP glucose-4-epimerase and UDP glucose-6-dehydrogenase respectively. **(b)** The relative expression pattern of differential expressed sugar transport genes. ov, ovule; ow, ovary wall/pericarp.

Regulation of carbohydrate metabolism is crucial for early fruit development. During cell division and cell expansion periods, sugar could be supplied as energy source and building blocks of cell wall as well^34^. Insufficient sugar supply by stress would cause severe fruit abortion^35^. To elucidate the spatial and temporal regulation network of carbohydrate metabolism, we isolated the genes involved in sugar metabolism and sugar transport among all differential expressed genes based on published information^34^,^36^ and the Sol Genomics Network (SGN) annotation to analyze their expression profiles (Supplementary Table S10). Among sugar metabolism genes, a group of genes showed elevated expression level specifically in ovule at 2 DPA or 5 DPA including cell wall invertases (*Solyc10g083290, Solyc09g010090* and *Solyc10g085360*) and beta-amylase (*Solyc09g091030*) (Fig. 5a). These enzymes catalyzed the decomposition of sucrose or starch indicating the high level of energy demands during embryo development. Another group exhibiting high expression in pericarp after fertilization contained genes encoding sucrose synthases and genes involved in cell wall synthesis such as UDP glycosyltransferase (*Solyc08g006350, Solyc08g006410* and *Solyc07g043480*), UDP glucose-4-epimerase (*Solyc08g080570*) and UDP glucose-6-dehydrogenase (*Solyc03g115380*)^37^,^38^,^39^ (Fig. 5a). This result was consistent with the fact that the pericarp undergoes a rapid cell division period since 2 DPA that the cell wall synthesis should be activated. Besides that, an ADP-glucose pyrophosphorylase (*Solyc01g079790*) which was involved in starch synthesis was also enriched in pericarp at 5 DPA (Fig. 5a) indicating the accumulation of starch in it at early fruit developmental stage^9^. For sugar transporter genes, three *SWEET* family genes had a peak expression at 5 DPA in ovule while *STP1, STP16, SFP3, PMT8* and *PMT5* showed highest expression at 5 DPA in ovary wall (Fig. 5b). Our validation of *SFP3* and *SWEET1a* by RT-qPCR further confirmed our observation (Supplementary Fig. S7). The SWEET family proteins were thought to have sucrose transporter activity^36^ and the STP, SFP and PMT proteins were considered as hexose transporters^34^ suggesting the different regulation on sugar transport in ovule and ovary wall. The dynamic changes of these genes during fruit early development indicated that the main sugar metabolism process in pericarp was the synthesis of polysaccharides while the ovule underwent the decomposition of sucrose to hexoses.

### Fruit growth regulated by tissue specific genes with time-course expression pattern

**Figure 6:**
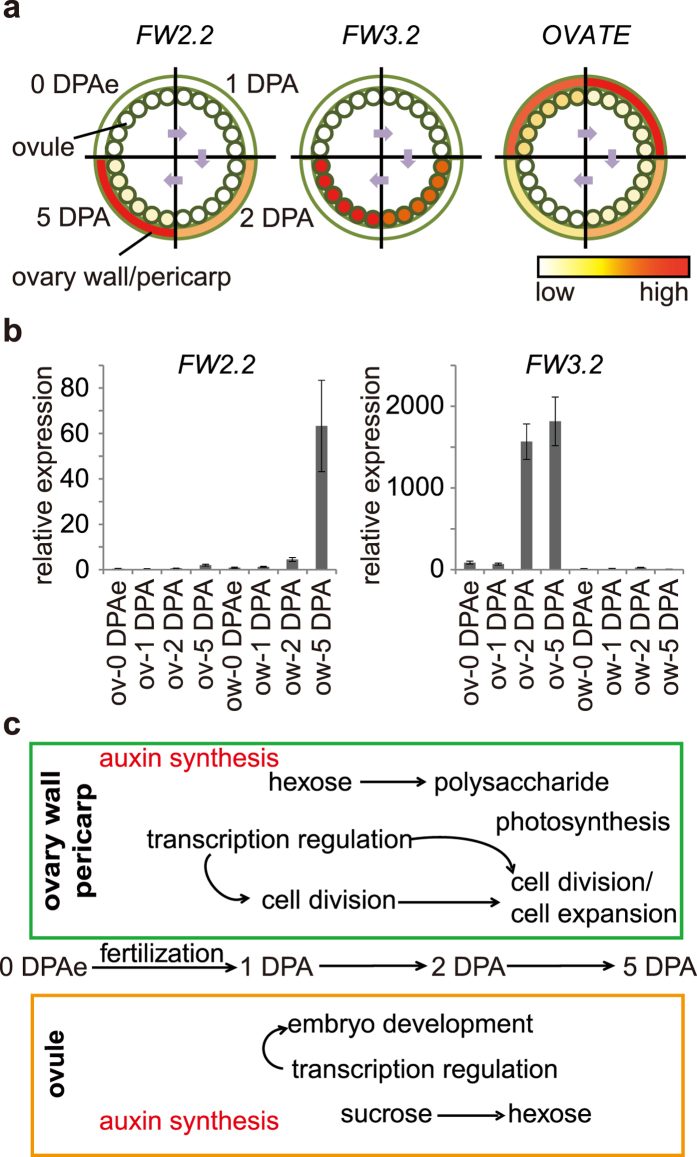
The tissue specific regulation of fruit development. (**a)** The schematic representation of spatiotemporal expression of *FW2.2, FW3.2* and *OVATE*. **(b)** The relative expression of *FW2.2* (left) and *FW3.2* (right) by RT-qPCR in different tissues and stages. The expression of each gene in ow-0 DPAe was normalized as 1. Error bars represent mean ± SEM. **(c)** The regulatory model for tomato early fruit development. ov, ovule; ow, ovary wall/pericarp.

In the history of domestication in tomato breeding, fruit size or mass has always been one of the most important traits to be selected. Early fruit development is crucial for the final weight of tomato fruit as it determines the number of cell layer in pericarp. In recent years, two domestication genes controlling fruit weight via regulating cell division, *FW2.2* and *FW3.2,* have been cloned^3^,^4^. Interestingly, we observed that *FW2.2* was mainly expressed in pericarp and *FW3.2* was enriched in ovule at early fruit developmental stages in an increasing manner according to our sequencing data (Fig. 6a). Their distinct expression pattern was validated by RT-qPCR (Fig. 6b). The other known gene controlling the fruit shape by affecting cell elongation is *OVATE*^40^, which was expressed in both ovule and ovary wall/pericarp but had a decrease manner during early fruit development (Fig. 6a). Taken together, these results indicate the signals from the ovule and the ovary wall were both important for regulating fruit traits.

According to our observation and previous studies, a set of genes including in diverse signals displayed spatial and temporal expression pattern to model the tomato fruit development (Fig. 6c). In the ovule and ovary wall/pericarp, distinct transcription factors and genes related with hormone and carbohydrate metabolism are drawn into various developmental stages. In the ovule, the sucrose was transported to the ovule and hydrolyzed to hexoses to provide the energy for embryonic development. In the pericarp, the hormones including auxin might induce cell division at 2 DPA and then the hexoses are transported to the ovary wall for cell wall biosynthesis and energy supply or storage. At the cell expansion stage, the photosynthesis is activated which provided more organic substance for fruit. Our model presents the sequential and specific transcriptional regulation during early fruit development.

## Discussion

After fertilization and fruit set, the fruit undergoes a complex developmental process until maturation. During this process, a lot of signals and metabolism pathways are involved via a developmental dependent manner^35^,^41^. In our experiment, more than 7,000 DEGs among stages present valuable information on genes expression profiles especially in TFs and sugar related genes. Our analysis provides insights into the whole picture about the spatial and temporal signal distribution at early fruit developmental stages especially in a high temporal resolution.

### The dynamic expression of genes is important for fruit development

Based on our clustered DEGs, GO term analysis demonstrates that several metabolic and hormone signaling pathways are dynamically regulated with the fruit development. Auxin plays critical roles in tomato early fruit development. The dynamic regulation of auxin signal is crucial for fruit formation according to our results. We observed that the *YUCCA* genes, such as *ToFZY2* and *ToFZY5*, are expressed not only with tissue-specific pattern but also in a time course manner with fruit development (Supplementary Fig. S6a). As the important enzymes in auxin biosynthesis, their transcripts are positively related with auxin accumulation^42^. The dynamic changes of these genes in our research provide new insights into the auxin accumulation in early fruit development.

Carbohydrate metabolism was also dynamically regulated during fruit development. Several cell wall invertase genes were highly expressed at 2 DPA or 5 DPA in ovule (Fig. 5a). Their expression patterns matched with the metabolic process during fruit early developmental stages in which hexoses including glucose and fructose accumulated^8^. Moreover, other genes involved in cell wall and starch synthesis showed higher expression at 5 DPA in pericarp (Fig. 5a), consistent with the accumulation of starch in pericarp at early fruit developmental stages^43^. Interestingly, we found genes showed elevated expression level at 2DPA and 5 DPA in pericarp were assigned to ‘photosynthesis’ (Fig. 2c,e), which in pericarp actually plays very limited role in fruit development but is essential for proper seed development^44^. This was correlated with our results that several *SWEET* family genes encoding sucrose transporters were highly expressed at 5 DPA in the ovule (Fig. 5b) which may transport carbohydrate synthesized in pericarp to the ovule for energy supply.

We also identified TFs showed spatial and temporal expression patterns during fruit development (Supplementary Fig. S2). After fertilization, there are 69 TFs showed elevated expression level in the ovary wall/pericarp at 2 DPA and 5 DPA (cluster 25 and 23, Supplementary Table S11). This is much more than the number (38) in the ovule (cluster 18 and 27, Supplementary Table S11). Our results showed opposite trend against the previous study in strawberry in which they identified 39 TFs highly expressed in embryo after fertilization and 24 TFs in ovary wall^12^. This difference between tomato and strawberry may be due to the distinct developmental processes of fruit in these two species. In tomato, the ovary wall develops to pericarp which forms the main part of the fleshy fruit. However, the real fruit in strawberry is the achene and the ovary wall barely develops after fertilization. So it is reasonable that the transcriptional regulation in tomato ovary wall/pericarp were much more complicated than that in strawberry.

### The specific expressed genes in pericarp provide new perspective to understand its development

The pericarp of tomato fruit mainly contributes to the fruit size and weight and further influences yield. During tomato domestication, the cultivated tomato fruit become more than 100 times larger than its ancestor^45^. An obvious difference is that the pericarp is thicker in cultivated tomato. By now, the genes controlling fruit size and weight, such as *FW2.2* and *FW3.2*, by affecting the cell number of pericarp have been cloned^3^,^4^. Interestingly, our observation showed that *FW2.2* expressed preferentially in pericarp and reached a peak expression at 5 DPA (Fig. 6a,b), suggesting that the genes specifically expressing in ovary wall/pericarp may be important for fruit development in tomato.

In our study, we detected the pericarp-specific expressed genes during early fruit developmental stages, which is distinct from other previous reports. There are 431 genes display pericarp-specific expression pattern which is much more than in ovule (Supplementary Table S7). Among them, the number of ovary wall/pericarp specific TFs were also more than two fold to that of ovule specific ones (Supplementary Table S9) suggesting more complicated transcriptional regulation and developmental processes in pericarp than in ovule, especially at early fruit developmental stages. One of the mainly enriched TFs in ovary wall/pericarp is the TALE family. Though TALE family members were found to regulate organ patterning in *Arabidopsis*, especially in the fruit^46^, their functions in fleshy fruit development are still unknown. In *Arabidopsis*, OFP1 could form a protein complex with KNAT6 and BLH1, which belong to TALE family, to regulate cell elongation^47^,^48^. We found that *Solyc05g005090* and *Solyc06g074120* are the homologues of Arabidopsis *KNAT6* and *BLH1*, which specifically expressed in the ovary wall/pericarp (Fig. 4b). Interestingly, *OVATE* of tomato shared a similar expression pattern with *Solyc05g005090* and *Solyc06g074120* in the ovary wall/pericarp according to our results (Fig. 6a, Supplementary Fig. S10), suggesting that the proteins encoded by *Solyc05g005090* and *Solyc06g074120* may suppress cell elongation in ovary wall/pericarp through interaction with OVATE. Overall, our results provided new candidate TFs which may participate in regulating early fruit growth and formation in tomato.

Previous studies indicated that the auxin synthesized in embryo will be transported into pericarp to promote fruit set at the early fruit developmental stage^24^, and several auxin biosynthesis genes are also mainly expressed in embryo at the specific fruit developmental stages^11^,^12^. With our spatiotemporal transcriptomes, we found that several auxin biosynthesis genes were specifically expressed in pericarp during early fruit development (Supplementary Fig. S6a), suggesting that *in situ* auxin biosynthesis is activated in it. Furthermore, carbohydrate metabolism is also specifically regulated in pericarp during early fruit development. Several genes including UDP glycosyltransferase, UDP glucose-4-epimerase and UDP glucose-6-dehydrogenase, which are involved in cell wall synthesis^37^,^38^,^39^, were specifically expressed in pericarp after fertilization (Fig. 5a). Besides that, an ADP glucose pyrophosphorylase which was involved in starch synthesis^43^ was also highly expressed in pericarp after fertilization (Fig. 5a) consistent with the accumulation of starch in pericarp during early fruit development^43^. Taken together, all these results suggested that genes specifically and temporally expressed in ovary wall/pericarp are important for fruit development in tomato. The future analysis on functions of ovary wall/pericarp specific genes may provide us insights on early fruit development in tomato and also uncover the new candidate genes controlling agricultural traits of tomato fruit in breeding.

## Methods

### Plant Material

Tomato plants were grown on soil in greenhouse. For samples at 1 DPA, 2 DPA and 5 DPA, the opening flowers were marked and the ovaries and little fruits were collected at the corresponding time. Samples of 0 DPAe were emasculated at –2 DPA first and collected at 0 DPA. The ovule and the ovary wall/pericarp was dissected under stereoscope and frozen in liquid nitrogen for further use. Samples for paraffin embedding were fixed in FAA (3.7% formaldehyde, 5% glacial acetic acid and 50% ethanol) and stored at room temperature for further embedding.

### Histology

The fixed samples were dehydrated in a graded series of ethanol (70%, 85%, 95% and 100%), followed by a xylene/ethanol series (xylene/ethanol 1:3, 1:1, 3:1 and 100% xylene). Xylene was replaced gradually with paraffin (Paraplast Plus, Sigma, P3683) at 60 °C for two days with four times replacement of paraffin. Ten μm sections were made using a HEISTION ERM3000 microtome and stained with Toluidine Blue O.

### RNA extraction

The RNA was extracted using TRIzol reagent (Invitrogen, 15596–026). The contaminant DNA was removed using TURBO DNA-free™ Kit (Ambion, AM1907).

### Library Construction and Sequencing

Briefly, RNA was fragmented first and reverse transcribed to cDNA by random primers. The strand-specific libraries were then constructed using dUTP method^49^. By this principle, the transcript direction and strand-specific information was reserved. Then the RNA-Seq libraries were sequenced separately on the Illumina HiSeq 2500 platform.

### RNA-seq Data Analysis

The RNA-seq reads were aligned to the tomato genome (ITAG2.4) using tophat-2.0.11^50^ with anchor length more than 8 nt for spliced alignments. Only uniquely mapped reads were retained for subsequent analysis. The expression levels for gene models from ITAG2.4 were measured and normalized as fragments per kilobase of transcript per million mapped reads (FPKM)^51^. Read coverage over gene body was calculated by RSeQC^52^ and the corresponding plot figure was made using ggplot2 with R script.

The statistical package DEGseq with MA-plot-based method in R version 3.0.3 was used to calculate *P* value that was adjusted using Benjamini-Hochberg procedure^53^. The fold change between two libraries was calculated by FPKM. Finally, fold change >3 and Benjamini-Hochberg adjusted *P* value < 0.001 were considered to be the threshold for identification of the differentially expressed genes (DEGs). The co-expression clustering of DEGs was performed using GENE-E (www.broadinstitute.org/cancer/software/GENE-E/) with normalized FPKM (FPKM/(FPKM_mean_ + 0.0001) − 1) and one minus Pearson correlation as the distance metric.

GO analysis of clustered genes and tissue specific genes was performed using their best homologous genes in *Arabidopsis* with DAVID (The Database for Annotation, Visualization and Integrated Discovery, https://david.ncifcrf.gov/)^54^.

### Quantitative reverse transcription polymerase chain reaction (RT-qPCR)

The reverse transcription was performed with TransScript II First-Strand cDNA Synthesis SuperMix (Transgen, AH301-02) using 2 μg total RNA. The qPCR was performed with RealSYBR Mixture (CWBIO, cw0760) on Bio-rad CFX-96 Real-time PCR Instrument. *UBI3* (*Solyc01g056840*) was used as the internal control of RT-qPCR. The relative expression of each gene in the ovary wall sample at 0 DPAe (ow-0 DPAe) was normalized as 1. Primers for RT-qPCR are listed in Supplementary Table S12.

### Data Deposition

The data discussed in this publication have been deposited in NCBI’s Gene Expression Omnibus^55^ and are accessible through GEO Series accession number GSE72216 (http://www.ncbi.nlm.nih.gov/geo/query/acc.cgi?acc=GSE72216). The accession number of individual library was listed in Supplementary Table S1.

## Additional Information

**How to cite this article**: Zhang, S. *et al*. Spatiotemporal transcriptome provides insights into early fruit development of tomato (*Solanum lycopersicum*). *Sci. Rep.* **6**, 23173; doi: 10.1038/srep23173 (2016).

## Supplementary Material

Supplementary Information

Supplementary Dataset 1

Supplementary Dataset 2

Supplementary Dataset 3

Supplementary Dataset 4

Supplementary Dataset 5

Supplementary Dataset 6

Supplementary Dataset 7

Supplementary Dataset 8

Supplementary Dataset 9

Supplementary Dataset 10
