# Supplementary material for: Spatiotemporal transcriptome provides insights into early fruit development of tomato (Solanum lycopersicum)
Source: Sci Rep. 2016 Mar 18;6:23173. doi: 10.1038/srep23173 (PMC4796798; doi:10.1038/srep23173)
Supplement: Supplementary Information [file srep23173-s1.pdf]

Spatiotemporal transcriptome provides insights into early fruit development of tomato (*Solanum lycopersicum*)

Shuaibin Zhang[1,a], Meng Xu[1,a], Zhengkun Qiu[a], Ketao Wang[a], Yongchen Du[a], Lianfeng Gu[b,*], Xia Cui[a,*]

a Key Laboratory of Biology and Genetic Improvement of Horticultural Crops of the Ministry of Agriculture, Sino-Dutch Joint Laboratory of Horticultural Genomics, The Institute of Vegetables and Flowers, Chinese Academy of Agricultural Sciences, Beijing 100081, China;

b Haixia Institute of Science and Technology (HIST), Fujian Agriculture and Forestry University, Fuzhou 350002, China.

1 These Authors contribute equally in this work.

* Correspondence: Xia Cui (cuixia@caas.cn), Lianfeng Gu (lfgu@fafu.edu.cn)

Supplementary Figure legends

Supplementary Figure S1. The relative expression pattern of all differential expressed genes.

Supplementary Figure S2. Identification of differential expressed transcription factors (TFs).

Supplementary Figure S3. The comparison between this study and the previous work (Pattison et al. 2015).

Supplementary Figure S4. Co-expression patterns of all clustered genes.

Supplementary Figure S5. RT-qPCR validation of ovule and ovary wall specific genes.

Supplementary Figure S6. The global expression pattern of auxin related genes.

Supplementary Figure S7. RT-qPCR validation of auxin and sugar related genes.

Supplementary Figure S8. The global expression pattern of GA related genes.

Supplementary Figure S9. The global expression pattern of cytokinin related genes.

Supplementary Figure S9. The comparison of read coverage along gene body between Pattison's data and data in this study.

Supplementary Figure S10. The schematic representation of spatiotemporal expression of *Solyc05g005090* and *Solyc06g074120*.

Supplementary Table legends

Supplementary Table S1. The mapping statistics of the RNA-seq datasets.

Supplementary Table S2. The genes expression in each sample.

Supplementary Table S3. The expression, fold change and adjusted *P* value of all differential expressed genes (DEGs).

Supplementary Table S4. The expression of transcription factors among differential expressed genes.

Supplementary Table S5. The comparison of the differential expressed genes (DEGs) in pericarp between this study and the previous work (Pattison *et al*. 2015).

Supplementary Table S6. The co-expression clusters of all differential expressed genes (DEGs).

Supplementary Table S7. The expression of ovule and ovary wall/pericarp specific genes in each sample.

Supplementary Table S8. The expression of auxin, GA and cytokinin related genes in each sample.

Supplementary Table S9. The expression of ovule and ovary wall/pericarp specific transcription factors in each sample.

Supplementary Table S10. The expression of sugar related genes among differential expressed genes.

Supplementary Table S11. The transcription factors in co-expression clusters.

Supplementary Table S12. The primers sequence used for RT-qPCR.

Fig. S1

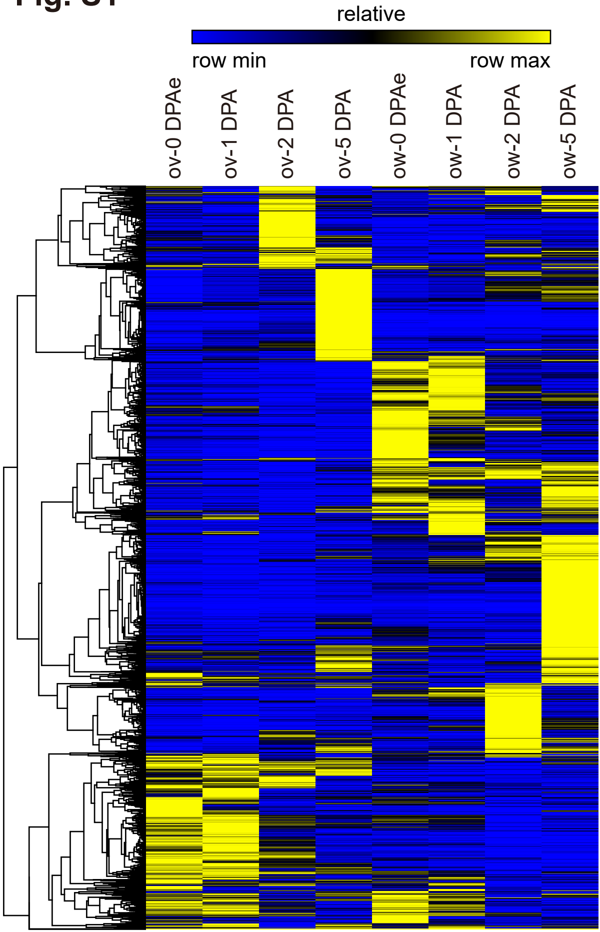

Supplementary Figure S1. The relative expression pattern of all differential expressed genes. ov, ovule; ow, ovary wall/pericarp.

Fig. S2

a

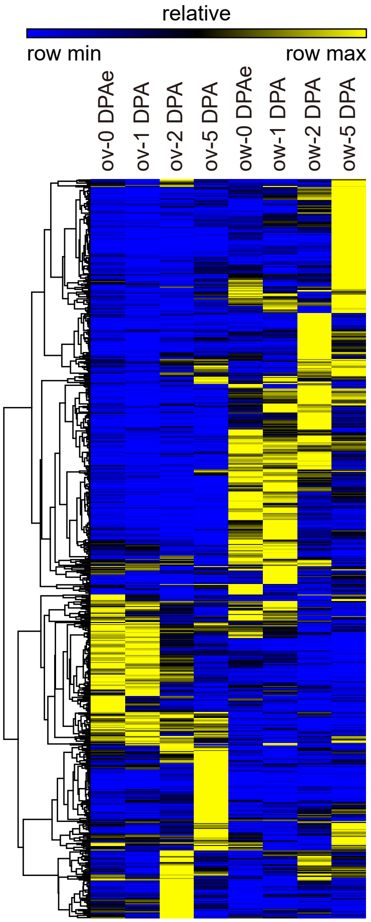

b

GO Term	*P*-value
regulation of transcription	0
transcription	1.01E-201
regulation of transcription, DNA-dependent	4.86E-139
regulation of RNA metabolic process	1.80E-138
response to organic substance	3.06E-41
response to ethylene stimulus	1.58E-36
response to endogenous stimulus	1.92E-33
response to hormone stimulus	1.01E-32
ethylene mediated signaling pathway	5.52E-25
response to chitin	1.08E-24
cellular response to hormone stimulus	3.76E-24
hormone-mediated signaling	3.76E-24
two-component signal transduction system (phosphorelay)	2.30E-23
flower development	3.18E-22
response to carbohydrate stimulus	2.92E-21
post-embryonic development	4.48E-19
intracellular signaling cascade	2.96E-15
reproductive structure development	8.33E-15
reproductive developmental process	2.80E-14
response to gibberellin stimulus	3.13E-13

Supplementary Figure S2. Identification of differential expressed transcription factors (TFs). (a) The relative expression pattern of differential expressed TFs. **(b)** The enriched GO terms in biological processes of differential expressed TFs. ov, ovule; ow, ovary wall/pericarp.

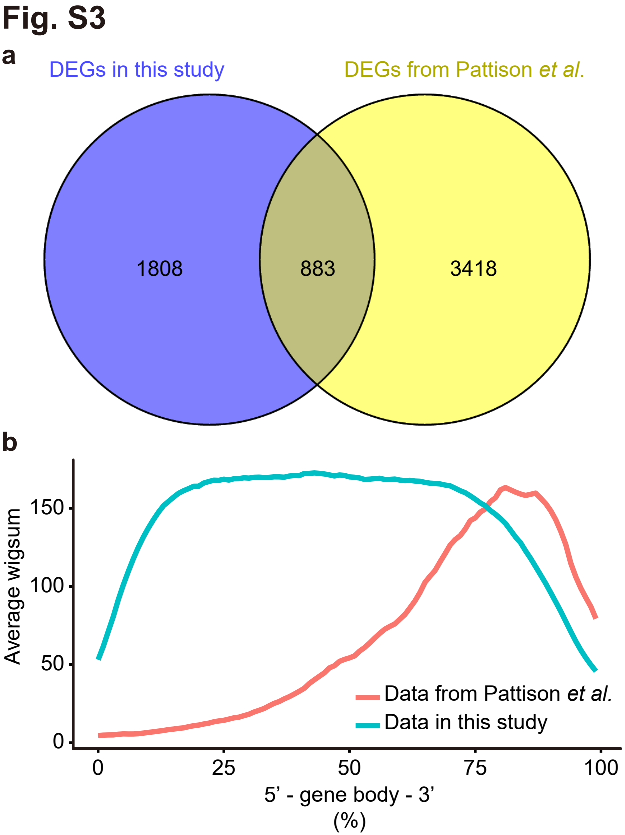

Supplementary Figure S3. The comparison between this study and the previous work (Pattison *et al*. 2015). (a) The differential expressed genes (DEGs) in pericarp between 0 DPAe and 5 DPA in this study and 0 DPA and 4 DPA in the previous work. **(b)** The read coverage along gene body from Pattison's data and data in this study.

Fig. S4

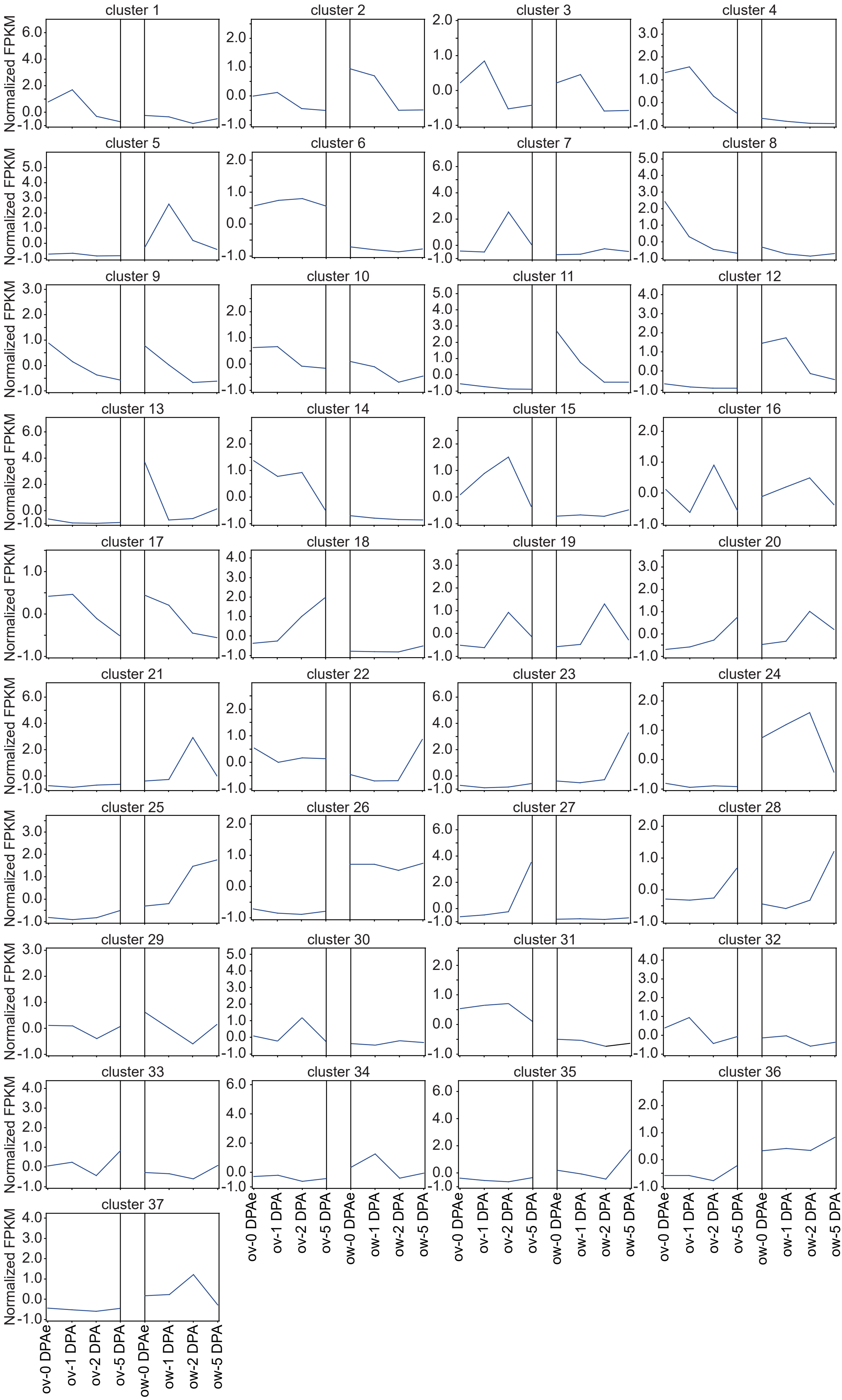

Supplementary Figure S4. Co-expression patterns of all clustered genes. The grey lines show the normalized FPKM of individual genes. The blue lines indicate the average of all genes within a cluster. ov, ovule; ow, ovary wall/pericarp.

Fig. S5

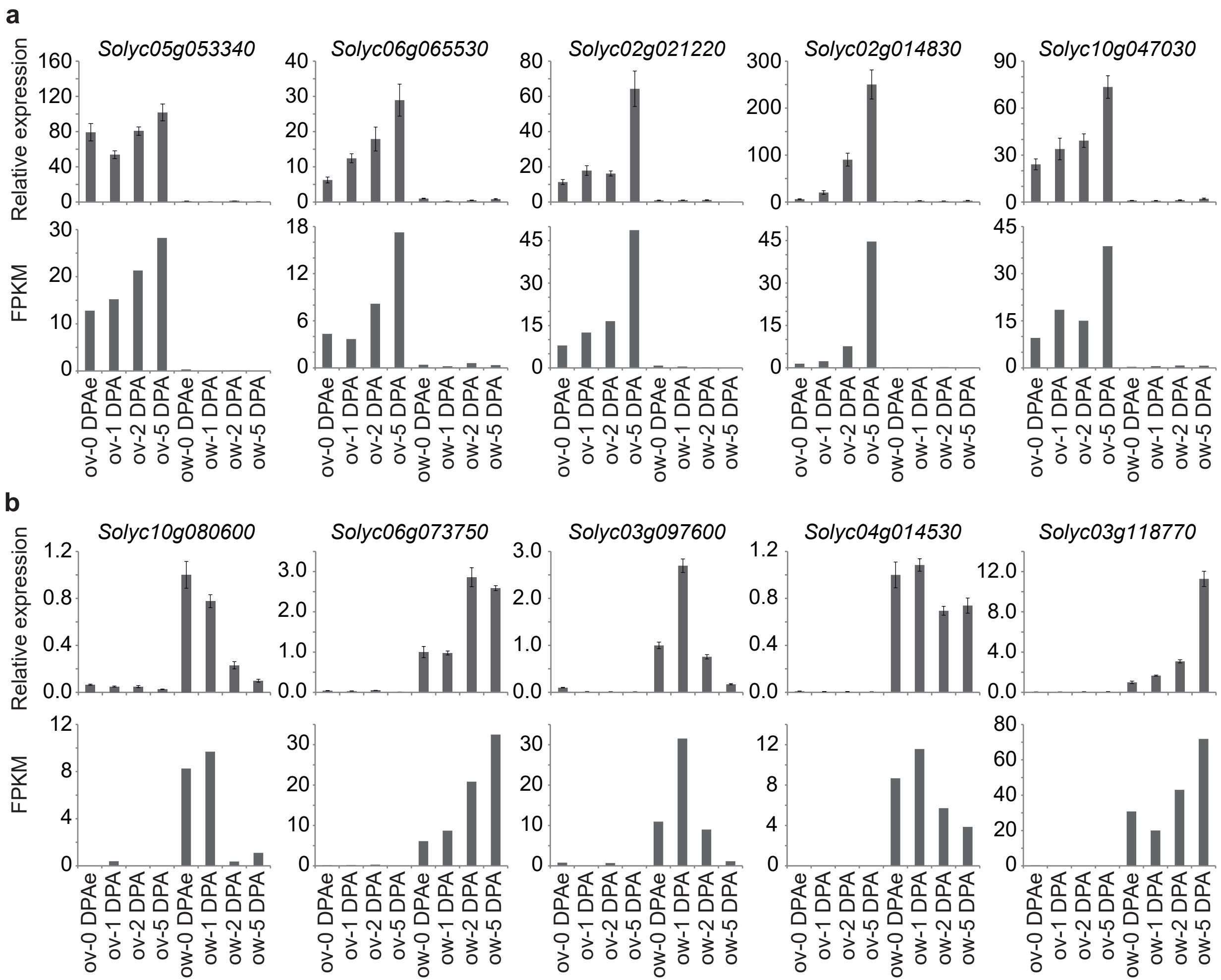

Supplementary Figure S5. RT-qPCR validation of ovule and ovary wall specific genes. (a) RT-qPCR (upper panel) validation and the average FPKM of two RNA-seq replicates (lower panel) of five ovule specific genes. **(b)** RT-qPCR (upper panel) validation and the average FPKM of two RNA-seq replicates (lower panel) of five ovary wall specific genes. ov, ovule; ow, ovary wall/pericarp. Error bars represent mean ± SEM.

Fig. S6

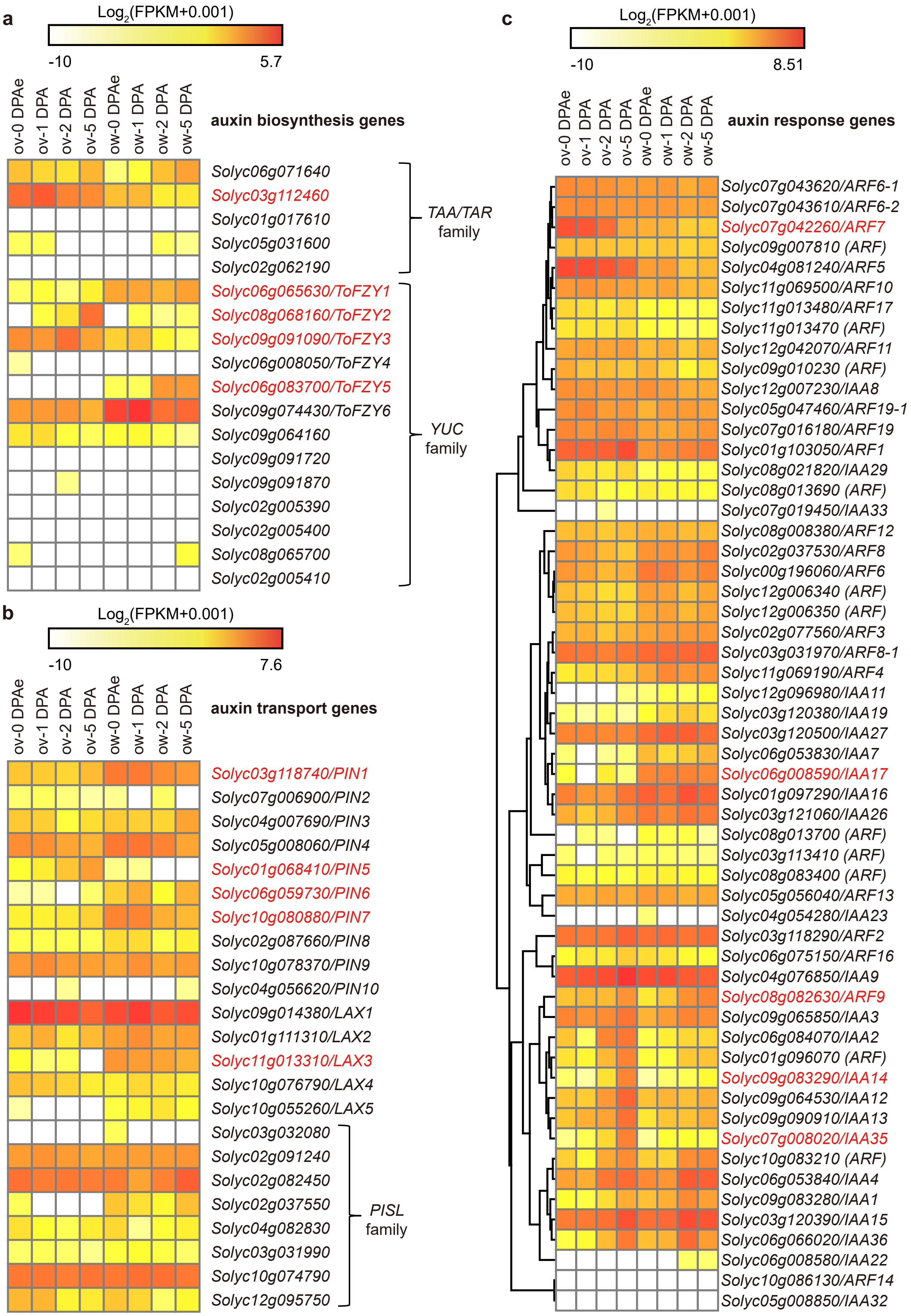

Supplementary Figure S6. The global expression pattern of auxin related genes. **(a)** The global expression pattern of auxin biosynthesis genes. **(b)** The global expression pattern of auxin transport genes. **(c)** The global expression pattern of auxin response genes. ov, ovule; ow, ovary wall/pericarp. Genes in red were mentioned in the main text.

Fig. S7

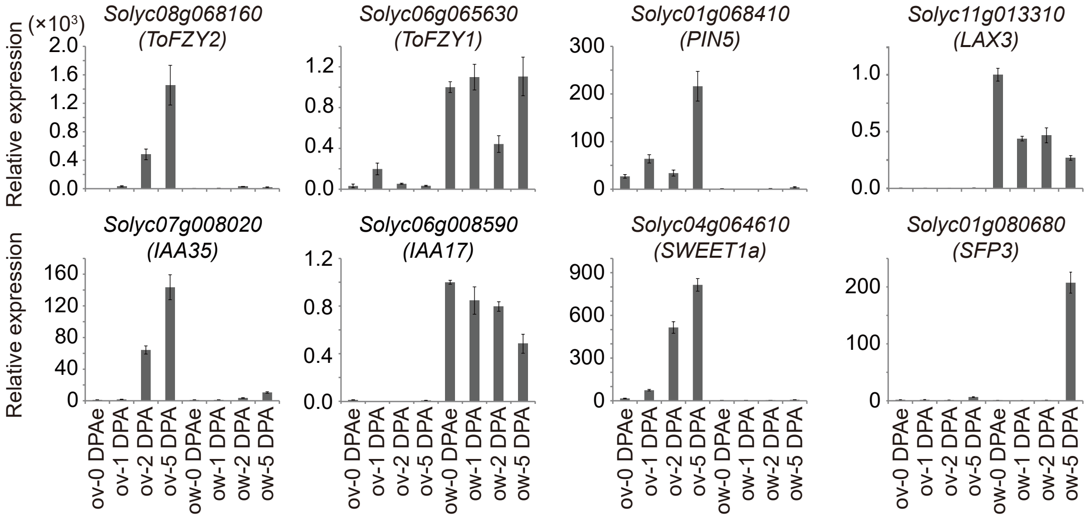

Supplementary Figure S7. RT-qPCR validation of genes involved in auxin biosynthesis (*ToFZY2* and *ToFZY1*), auxin transport (*PIN5* and *LAX3*), auxin response (*IAA35* and *IAA17*) and sugar transport (*SWEET1a* and *SFP3*) with tissue specific expression. The expression of each gene in ow-0 DPAe was normalized as 1. ov, ovule; ow, ovary wall/pericarp. Error bars represent mean ± SEM.

Fig. S8

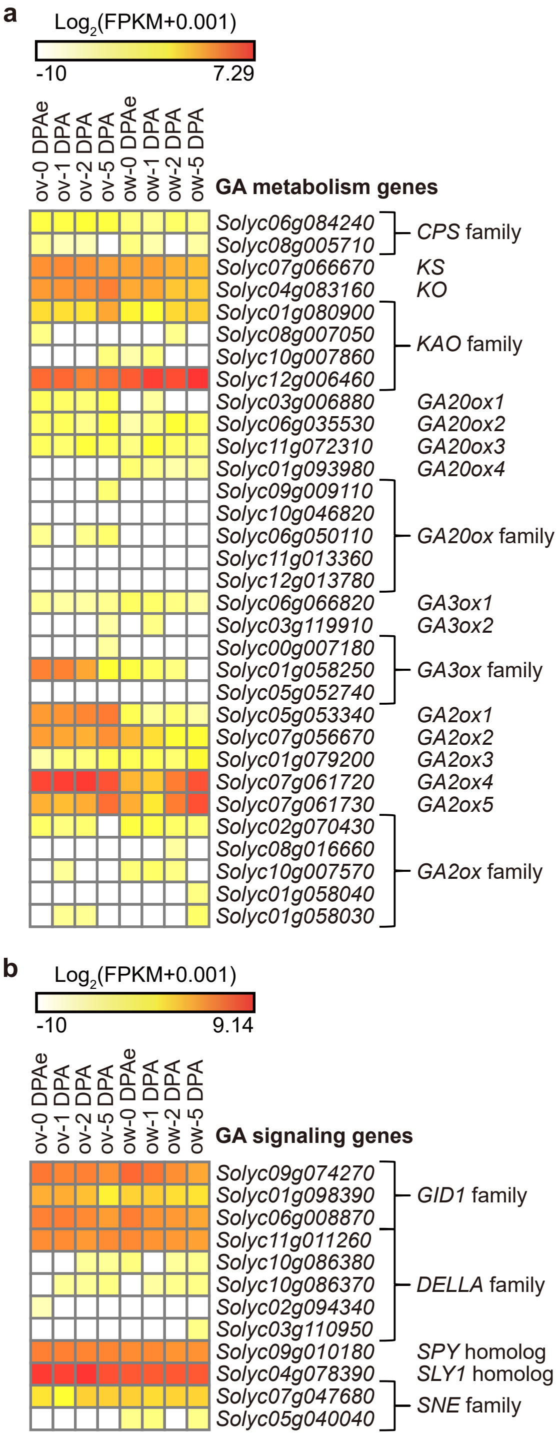

Supplementary Figure S8. The global expression pattern of GA related genes. (a) The global expression pattern of GA metabolism genes. **(b)** The global expression pattern of GA signaling genes. ov, ovule; ow, ovary wall/pericarp.

Fig. S9

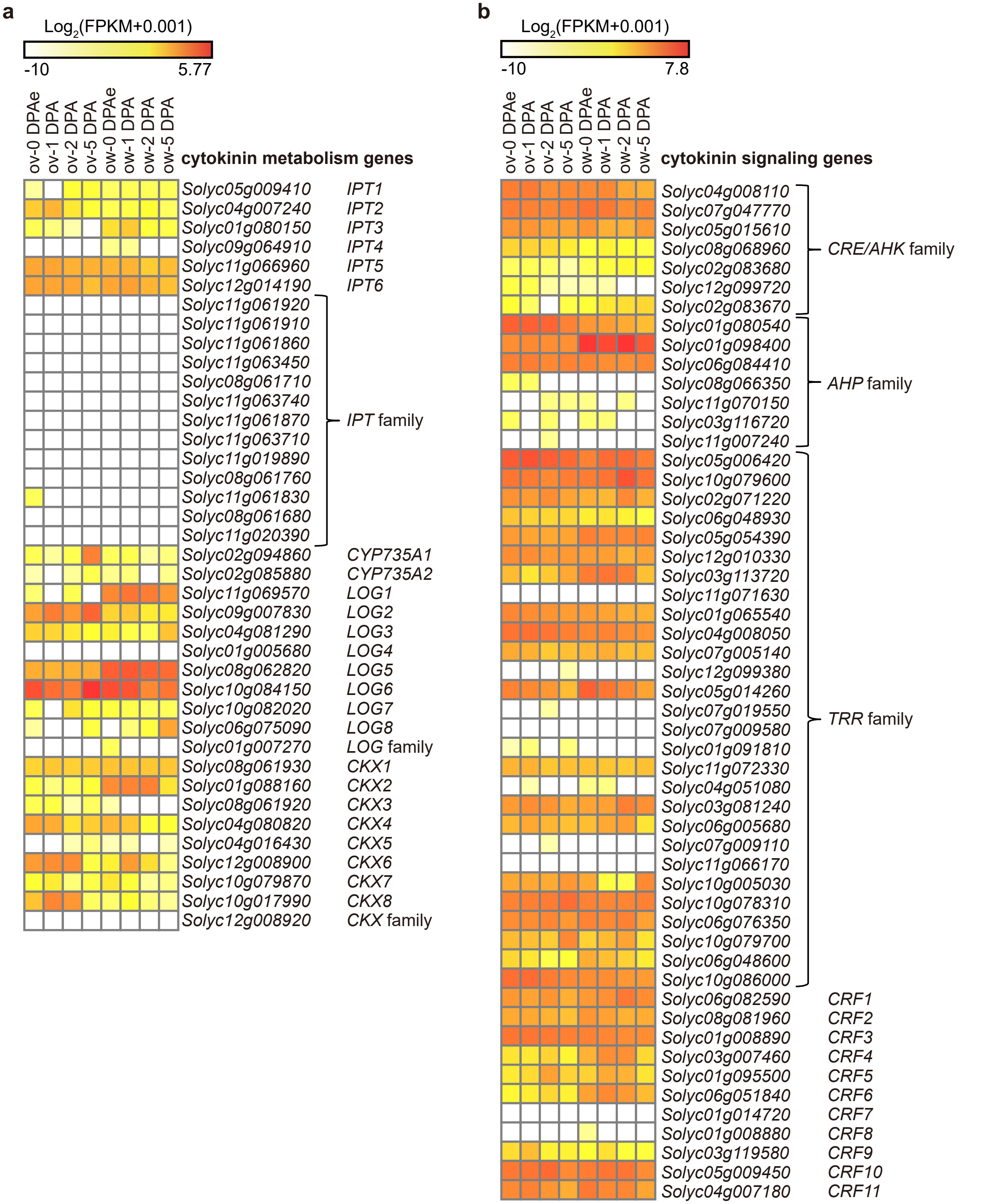

Supplementary Figure S9. The global expression pattern of cytokinin related genes. (a) The global expression pattern of cytokinin metabolism genes. **(b)** The global expression pattern of cytokinin signaling genes. ov, ovule; ow, ovary wall/pericarp.

Fig. S10

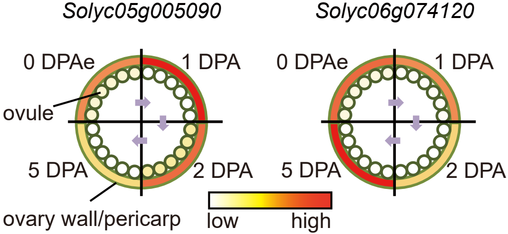

Supplementary Figure S10. The schematic representation of spatiotemporal expression of *Solyc05g005090* and *Solyc06g074120*.

Supplementary Table S1. The mapping statistics of the RNA-seq datasets. ov, ovule; ow, ovary wall/pericarp; DPA, days post anthesis; e, emasculation.

Library	Accession number	Total fragments	Total match fragments[a]	Unique match fragments[b]	Multiple match fragments[c]	Correlation between replicates (R^2)
ov-0DPAe rep1	GSM1857771/SRX1160117	22652386	20851533 (92.05%)	20462188 (98.13%)	389345 (1.87%)	0.9491
ov-0DPAe rep2	GSM1857772/SRX1160118	25272927	23368895 (92.47%)	22990451 (98.38%)	378444 (1.62%)	
ov-1DPA rep1	GSM1857767/SRX1160113	18973906	17787285 (93.75%)	17482452 (98.29%)	304833 (1.71%)	0.9477
ov-1DPA rep2	GSM1857768/SRX1160114	22515268	19830128 (88.07%)	19469416 (98.18%)	360712 (1.82%)	
ov-2DPA rep1	GSM1857775/SRX1160121	20806844	17390918 (83.58%)	17115446 (98.42%)	275472 (1.58%)	0.9679
ov-2DPA rep2	GSM1857776/SRX1160122	22276864	19927138 (89.45%)	19574875 (98.23%)	352263 (1.77%)	
ov-5DPA rep1	GSM1857779/SRX1160125	19692795	17891403 (90.85%)	17673799 (98.78%)	217604 (1.22%)	0.9076
ov-5DPA rep2	GSM1857780/SRX1160126	21350247	19640238 (91.99%)	19343758 (98.49%)	296480 (1.51%)	
ow-0DPAe rep1	GSM1857773/SRX1160119	21349158	19560342 (91.62%)	19124983 (97.77%)	435359 (2.23%)	0.9281
ow-0DPAe rep2	GSM1857774/SRX1160120	23587693	21702643 (92.01%)	21401246 (98.61%)	301397 (1.39%)	
ow-1DPA rep1	GSM1857769/SRX1160115	18448228	16674854 (90.39%)	16460019 (98.71%)	214835 (1.29%)	0.9281
ow-1DPA rep2	GSM1857770/SRX1160116	20551499	14014987 (68.19%)	13751323 (98.12%)	263664 (1.88%)	
ow-2DPA rep1	GSM1857777/SRX1160123	32267090	27069637 (83.89%)	26681092 (98.56%)	388545 (1.44%)	0.9736
ow-2DPA rep2	GSM1857778/SRX1160124	22126596	19939012 (90.11%)	19656929 (98.59%)	282083 (1.41%)	
ow-5DPA rep1	GSM1857781/SRX1160127	21751636	19631261 (90.25%)	19381995 (98.73%)	249266 (1.27%)	0.8875
ow-5DPA rep2	GSM1857782/SRX1160128	22417261	20114326 (89.73%)	19810042 (98.49%)	304284 (1.51%)	

a Number of fragments that aligned to tomato genome (ITAG2.4).

b Number of fragments that aligned to a unique location.

c Number of mapped fragments which have more than one genomic location.

Supplementary Table S12. The primers sequence used for RT-qPCR.

Gene	Primer name	Primer sequence
Solyc05g053340	XP486	AATCAGTGAGGGAGTTGGCA
	XP487	GGGTCCAGTTGAATGGAGGG
Solyc06g065630	XP548	GGCCCTTCTAAAATGGATGCC
	XP549	ATGCTCCAACGTCGAGTACC
Solyc02g021220	XP558	CAAGCCGGATGTGATTGCAC
	XP559	ACCAGTATGGGTGAGCTCCT
Solyc02g014830	XP560	TTCAAAGGATTCATGGAACTGC
	XP561	CACAATTGGGGGCGTAAAGG
Solyc10g047030	XP572	GTCTGGAGGTGGAATGGACG
	XP573	GGGTAGTTTGTGGCAGGGTT
Solyc10g080600	XP476	CTTCCCAACATCACAAGCCC
	XP477	TAACGCTGACAATGAACGGTG
Solyc06g073750	XP472	GTCGTCCAGTCGTGCTAGAG
	XP473	GTGGCTGAGTTGTGAGTCCA
Solyc03g097600	XP470	AGGGTGAAGTCATTGTGAAGGA
	XP471	GGTTTGCAACTTGGGCTCAT
Solyc04g014530	XP478	AGAGGTCACGTCTCATCACC
	XP479	AAGTGCCAATGGACCTCTCA
Solyc03g118770	XP480	ACGGCGAATCCAACGAATTG
	XP481	TCCAGCGTTTGCAGTTGTTC
Solyc08g068160/ ToFZY2	XP484	ACTTGACGTTGGAACGCTTG
	XP485	TTTCCTTTAGCCACGAGGGC
Solyc06g065630/ ToFZY1	XP554	GGCCCTTCTAAAATGGATGCC
	XP555	ATGCTCCAACGTCGAGTACC
Solyc01g068410/ PIN5	XP594	GGTCTCTTCTGGGCTCTCGT
	XP595	AAGTGCCATTGTTGCTGGAC
Solyc11g013310/ LAX3	XP466	GGGAGTGAAGCACTCAGGAC
	XP467	TTGGCAACAAAGCCAAAGCA
Solyc07g008020/ IAA35	XP482	CGTTAGTGGTGGCAGAGGAT
	XP483	GCACTGACCCAACAAAAGTCC
Solyc06g008590/ IAA17	XP598	TTAAGGCTAGGGTTGCCTGG
	XP599	AGCTGGGGTTTTGTGTGTTG
Solyc04g064610/ SWEET1a	XP488	TGCTGTAGCCCTTGTTTCCA
	XP489	CCAGGAAGCGCCACATAAGA
Solyc01g080680/ SFP3	XP462	ATGGTGTCAGCAACAGGGAC
	XP463	GTGTAAGAAACAGCCCACGC
Solyc02g090730/ FW2.2	XP442	CTGGGATTGACAGGATTGCCT
	XP443	TAGCTTGCCACCCTATTCCC
Solyc03g114940/ FW3.2	XP444	ATTACTCTCGTGGGCAAGGC
	XP445	CTGAACCGAATGGTGCAAGC
Solyc01g056940/ UBI3	XP157	TCTTCCGACACCATCGACAA
	XP158	AGAACTGCAACACAGTGAGC
